# Supplementary material for: B cell-derived GABA elicits IL-10+ macrophages to limit anti-tumour immunity
Source: Nature. 2021 Nov 3;599(7885):471–6. doi: 10.1038/s41586-021-04082-1 (PMC8599023; doi:10.1038/s41586-021-04082-1)
Supplement: Supplementary file 1 — This file contains Supplementary Fig. 1 (Sorting strategies for isolation of immune cells in the peripheral organs) and Fig. 2 (Sorting strategies for isolation of immune cells in tumour tissues). [file 41586_2021_4082_MOESM1_ESM.pdf]

nature portfolio

https://doi.org/10.1038/s41586-021-04082-1

Supplementary information

B cell-derived GABA elicits IL-10 macrophages to limit anti-tumour immunity

In the format provided by the authors and unedited

nature portfolio

https://doi.org/10.1038/s41586-021-04082-1

Supplementary information

B cell-derived GABA elicits IL-10 macrophages to limit anti-tumour immunity

In the format provided by the authors and unedited

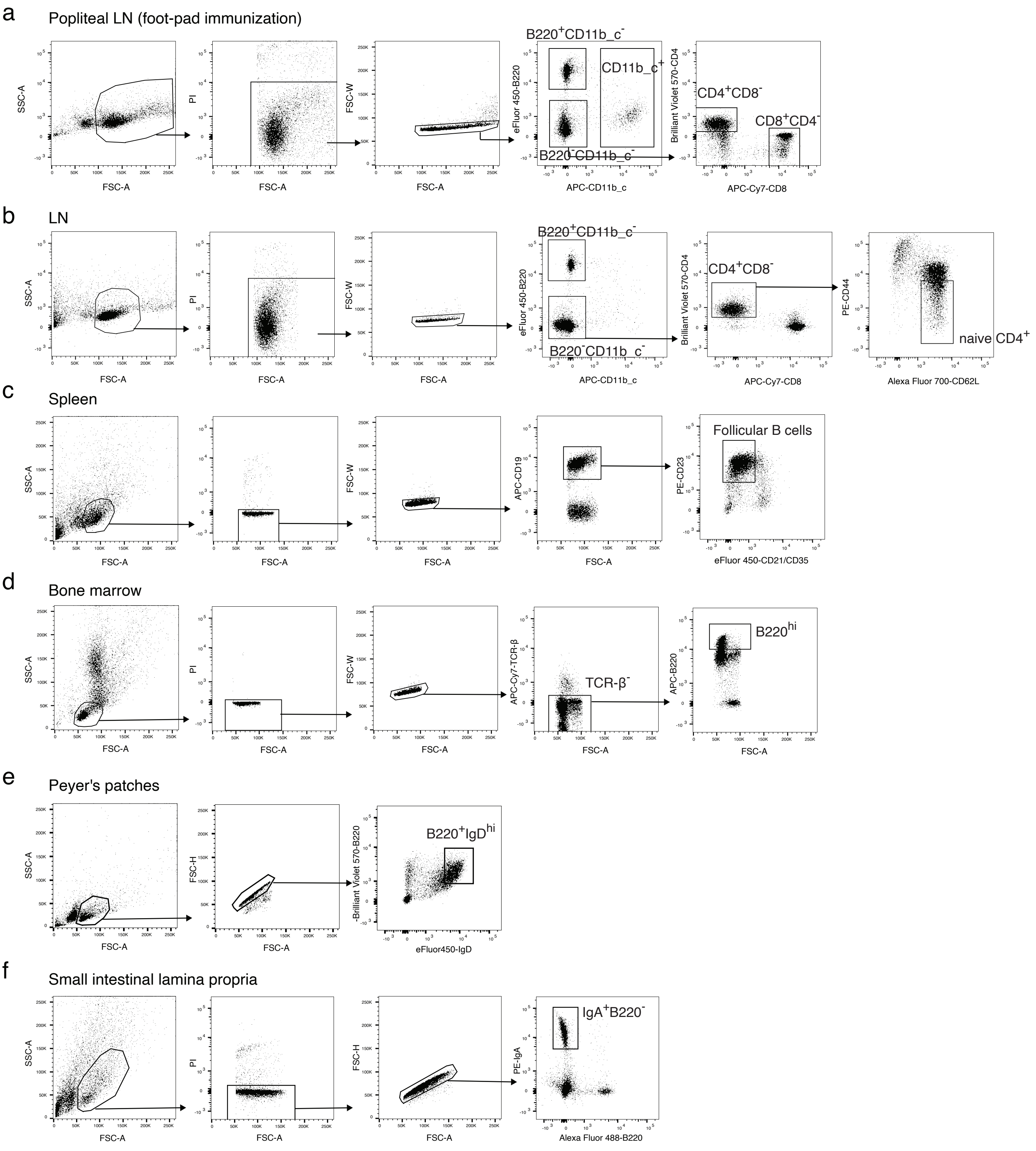

Supplementary Figure 1. Sorting strategies for isolation of immune cells in the peripheral organs

a, $CD4^+$ or $CD8^+$ T cells ($CD11c^-$, $CD11b^-$, $B220^-$, $CD4^+$ or $CD8^+$), B cells ($CD11c^-$, $CD11b^-$, $CD4^-$, $CD8^-$, $B220^+$) and Mf/DC ($B220^-$, $CD11c^+$ and/or $CD11b^+$) were sorted from popliteal LNs after footpad immunazation, in Fig. 2a. **b-f,** naïve $CD4^+$ T cells ($CD11c^-$, $CD11b^-$, $B220^-$, $CD8^-$, $CD4^+$, $CD44^{low}$, $CD62L^+$), B cells ($CD11c^-$, $CD11b^-$, $CD4^-$, $CD8^-$, $B220^+$) cells were sorted from LNs (pooled axillary, brachial and inguinal LNs) (**b**), follicular B cells ($CD19^+$, $CD21^{mid}$, $CD23^+$) were sorted from spleen (**c**), bone marrow B cells ($B220^{hi}$, $TCR\text{-}\beta^-$) (**d**), Peyer's patch B cells ($B220^+$, IgD^{hi}) (**e**) were sorted as defined, and lamina propria IgA plasma cells (IgA^+, $B220^-$) (**f**) were sorted from small intestine of WT mice, in Fig. 2b.

a

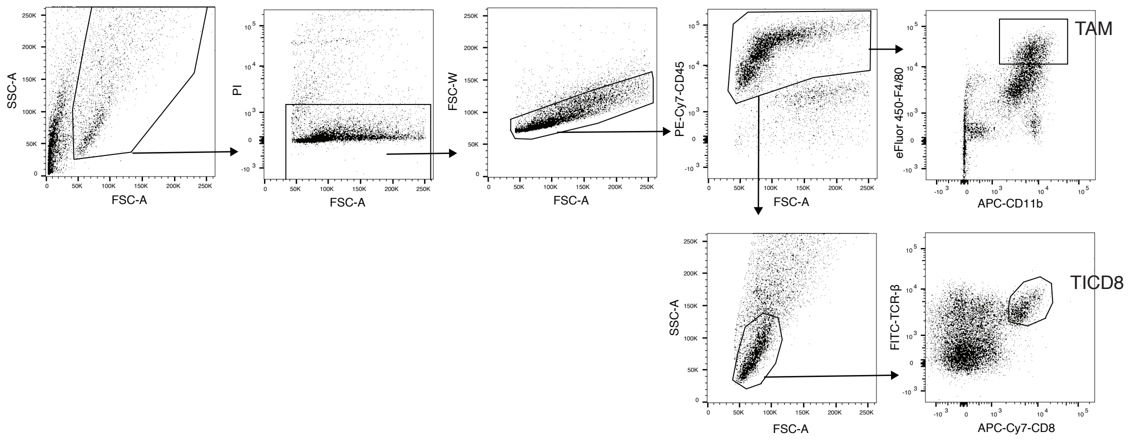

b

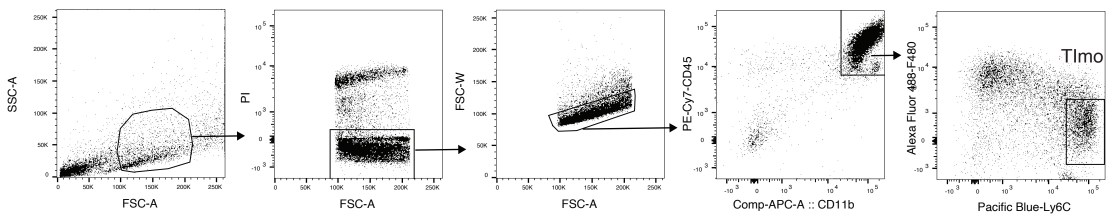

Supplementary Figure 2. Sorting strategies for isolation of immune cells in tumor tissues

a, Tumor-assosiated macrophages (TAMs) or tumor infiltrating $CD8^+$ T cells (TICD8) were sorted from tumor of WT or *muMt*$^{-/-}$ mice implanted with GABA or placebo pellets, or WT mice injected with DMSO or picrotoxin as shown in Fig. 3f, g, h, Extended Data Fig. 6d and Extended Data Fig. 10. **b,** Tumor infiltrating monocytes (TImo) were isolated from tumor of WT mice, in Fig. 4e.
