# Supplementary material for: B cell-derived GABA elicits IL-10+ macrophages to limit anti-tumour immunity
Source: Nature. 2021 Nov 3;599(7885):471–6. doi: 10.1038/s41586-021-04082-1 (PMC8599023; doi:10.1038/s41586-021-04082-1)
Supplement: Supplementary file 2 — Reporting Summary [file 41586_2021_4082_MOESM2_ESM.pdf]

nature portfolio

Corresponding author(s): Fagarasan Sidonia

Last updated by author(s): 2021/09/10

Reporting Summary

Nature Portfolio wishes to improve the reproducibility of the work that we publish. This form provides structure for consistency and transparency in reporting. For further information on Nature Portfolio policies, see our Editorial Policies and the Editorial Policy Checklist.

Statistics

For all statistical analyses, confirm that the following items are present in the figure legend, table legend, main text, or Methods section.

n/a	Confirmed	
☐	☒	The exact sample size (*n*) for each experimental group/condition, given as a discrete number and unit of measurement
☐	☒	A statement on whether measurements were taken from distinct samples or whether the same sample was measured repeatedly
☐	☒	The statistical test(s) used AND whether they are one- or two-sided<br>*Only common tests should be described solely by name; describe more complex techniques in the Methods section.*
☐	☒	A description of all covariates tested
☐	☒	A description of any assumptions or corrections, such as tests of normality and adjustment for multiple comparisons
☐	☒	A full description of the statistical parameters including central tendency (e.g. means) or other basic estimates (e.g. regression coefficient) AND variation (e.g. standard deviation) or associated estimates of uncertainty (e.g. confidence intervals)
☐	☒	For null hypothesis testing, the test statistic (e.g. *F*, *t*, *r*) with confidence intervals, effect sizes, degrees of freedom and *P* value noted<br>*Give P values as exact values whenever suitable.*
☒	☐	For Bayesian analysis, information on the choice of priors and Markov chain Monte Carlo settings
☐	☒	For hierarchical and complex designs, identification of the appropriate level for tests and full reporting of outcomes
☐	☒	Estimates of effect sizes (e.g. Cohen's *d*, Pearson's *r*), indicating how they were calculated

Our web collection on statistics for biologists contains articles on many of the points above.

Software and code

Policy information about availability of computer code

Data collection	Aria II flow cytometry system was used for collection of FACS data. LC-MS and HPLC (Ultimate3000 system) were used for collection of metabolite data. BZ-X700 fluorescence microscope was used for collection of photomicrograph. LightCycler 96 (SN: 12718) was used for collection q-PCR data. MiSeq System was used for collection RNA sequencing data. Q-Exactive Plus Orbitrap mass spectrometer with a Nanospray Flex ion source coupled to an EASY-nLC 1200 system was used for collection of proteome data
Data analysis	Flowjo software (10.7.1) was used for FACS analysis. BZ-X analyzer software was used for analysis of photomicrograph. Compound Discoverer 2.0 software was used for non-target analysis of mass spectrometry data. Proteome Discoverer (2.4) was used for analysis of proteome data. MetaboAnalyst 5.0 was used for analysis of metabolome data. LightCycler 96 SW 1.1 software was used for analysis of q-PCR data. DEseq2(ver. 1.30.1) was used for analysis of RNA sequencing data. Ingenuity Pathway Analysis (01-18-06) was used for the pathway and upstream regulator analysis of RNA sequencing and proteome data. PRISM 8 software was used for statistical analysis.

For manuscripts utilizing custom algorithms or software that are central to the research but not yet described in published literature, software must be made available to editors and reviewers. We strongly encourage code deposition in a community repository (e.g. GitHub). See the Nature Portfolio guidelines for submitting code & software for further information.

Data

Policy information about availability of data

All manuscripts must include a data availability statement. This statement should provide the following information, where applicable:

- Accession codes, unique identifiers, or web links for publicly available datasets
- A description of any restrictions on data availability
- For clinical datasets or third party data, please ensure that the statement adheres to our policy

Source data for quantifications represented in all graphs plotted in figures and extended data figures are available in the online version of the paper. The RNA-seq datasets analyzed are publicly available in the Gene Expression Omnibus repository with the accession numbers GSE169543 and GSE183246 (released on September 02, 2021). The Gene chip datasets are provided in Supplementary Table. The proteomics datasets are available via ProteomeXchange with identifier PXD028403 (released on the date of online publication). The DESeq2 (1.30.1) package was used for analyzing RNA-seq data (https://bioconductor.org/packages/release/bioc/html/DESeq2.html).

Field-specific reporting

Please select the one below that is the best fit for your research. If you are not sure, read the appropriate sections before making your selection.

☒ Life sciences ☐ Behavioural & social sciences ☐ Ecological, evolutionary & environmental sciences

For a reference copy of the document with all sections, see nature.com/documents/nr-reporting-summary-flat.pdf

Life sciences study design

All studies must disclose on these points even when the disclosure is negative.

Sample size	No sample size calculation was performed. The number of animals was determined based on the number of animals implemented in previously published papers. The number of human samples was determined based on availability. Each experiment was replicated for subsequent statistical analysis.
Data exclusions	Infrequently, mice showed signs of inflammation even in normal SPF condition. Therefore, when sacrificed, mice were routinely checked for the inflammation status and samples from mice showing severe inflammation status (splenomegaly and colitis) were excluded.
Replication	Each animal experiment was performed with at least 3 biological replicates. Clinical human experiment was performed with at least 3 biological replicates except for imaging MS. All attempts at replication gave similar results and reliably reproduced.
Randomization	Age and sex matched mice were randomly allocated into experimental groups. In human study, patients with RA were randomly recruited after informed consent and selected based on the availability of plasma sample, then patients together with symptoms other than RA (stroke, herpes zoster, dementia, cancer, hemodialysis, pneumonia, surgery in a year) and under steroid treatment were removed from the analysis to investigate pure RA effect on metabolites. Since ratio of male and female is 1:5.8 in the subject group, we focused on female to avoid sex bias.
Blinding	Blinding was performed in the measurement of the tumor size.<br>Otherwise blinding was not used since the data collection and the analysis were performed with quantitative instruments to maintaining objectivity.

Reporting for specific materials, systems and methods

We require information from authors about some types of materials, experimental systems and methods used in many studies. Here, indicate whether each material, system or method listed is relevant to your study. If you are not sure if a list item applies to your research, read the appropriate section before selecting a response.

Materials & experimental systems

n/a	Involved in the study	
☐	☒	Antibodies
☐	☒	Eukaryotic cell lines
☒	☐	Palaeontology and archaeology
☐	☒	Animals and other organisms
☐	☒	Human research participants
☐	☒	Clinical data
☒	☐	Dual use research of concern

Methods

n/a	Involved in the study	
☒	☐	ChIP-seq
☐	☒	Flow cytometry
☒	☐	MRI-based neuroimaging

Antibodies

Antibodies used	(Flow Cytometry)<br>APC-Cy7-anti-CD8a (Biolegend, clone 53-6.7, # 100713, 1:100), APC-anti-TCR-β (Biolegend, clone H57-597, # 109211, 1:50), Brilliant Violet 570-anti-CD4 (Biolegend, clone RM4-5, # 100542, 1:100), Alexa Fluor 700-anti-CD62L (Biolegend, clone MEL-14, # 104426, 1:100), APC-nti-CD11c (Biolegend, clone N418, 117309, 1:100), APC-anti-CD11b (Biolegend, clone M1/70, # 101211, 1:100), anti-CD3ε (Biolegend, clone 145-2C11), PE-Cy7-anti-CD45.2 (Biolegend, clone 104, #109829, 1:100), APC-anti-Granzyme B (Biolegend, clone QA16A02, # 372203, 1:100), PE-anti-Perforin (Biolegend, clone S16009B, # 154405, 1:100), Alexa Fluor 488-anti-F4/80 (Biolegend, clone BM8, # 123119, 1:100), FITC-anti-cKit (Biolegend, clone 2B8, #105805, 1:100), PE-Cy7-anti-SCA-1 (Biolegend, clone D7, #108113, 1:100), PE-anti-CD48 (Biolegend, clone HM48-1, # 103405, 1:100), Pacific Blue-anti-CD150 (Biolegend, clone TC15-12F12.2, # 115923, 1:100), PE-Cy7-anti-CD93 (Biolegend, clone AA4.1, #136505, 1:200), PE-Cy7-anti-CD38 (Biolegend, clone 90, # 102718, 1:100), APC-anti-IFN-γ (eBioscience, clone XMG1.2, #16-7311, 1:100), PE-anti-CD44 (eBioscience, clone IM7, #12-0441, 1:100), APC-anti-B220 (eBioscience, clone RA3-6B2, # 17-0452, 1:100), eFluor450-anti-B220 (eBioscience, clone RA3-6B2, # 48-0452,1:100), Brilliant Violet 570-anti-B220 (Biolegend, clone RA3-6B2, #103237, 1:100), Alexa Fluor 488-anti-B220 (eBioscience, clone RA3-6B2, # 53-0452, 1:100), PerCP eFluor710-anti-TNF-α (eBioscience, clone MP6-XT22, # 46-7321, 1:100), eFluor450-anti-IgD (eBioscience, clone 11-26c, #48-5993, 1:200), eFluor 450-anti-CD21/CD35 (eBioscience, clone eBio4E3, # 48-0212, 1:200), anti-FOXP3 (eBioscience, clone FJK-16s), PE-anti-CD25 (BD Biosciences, clone PC61, # 553866, 1:50), PE-anti-IL-2 (BD Biosciences, clone JES6-5H4, # 554428, 1:100), PE-anti-CD23 (BD Biosciences, clone B3B4, # 553139, 1:200), PE-Cy7-anti-CD43 (BD Biosciences, clone S7, # 01605B, 1:100, FITC-anti-CD16/32 (BD Biosciences, clone 2.4G2, # 553144, 1:100), APC-anti-CD19 (BD Biosciences, clone 1D3, #550992, 1:100), anti-CD5 (BD Biosciences, clone 53-7.3), anti-CD95 (BD Biosciences, clone Jo2), anti- γδ TCR (BD Biosciences, clone GL3), FITC-anti-IgM (SouthernBiotech, polyclonal, #1022-02, 1:100) and PE-anti-IgA (SouthernBiotech, polyclonal, # 1040-09, 1:200).<br><br>(Sorting)<br>biotin-anti-CD20 (Biolegend, clone 2H7, #302349, 1:100) , biotin-anti-CD19 (Biolegend, clone HIB19, # 302203, 1:100)<br><br>(Culture) concentration as described in Method<br>anti-CD3 (BD Biosciences, 145-2C11, #567115)<br>anti-CD28 (BD Biosciences, 37.51, # 553294)<br>anti-IgM (Jackson ImmunoReserch,)<br>anti-CD40 (BD Biosciences,3/23 or HM40-3, # 553787 or 553721)<br>F(ab')2-Goat anti-human IgG/IgM (Invitrogen,)<br>anti-IL-10 blocking antibodies (eBioscience, JES5-2A5, # 16-7102)<br>(Imaging of NFkB)<br>anti-human total p65 rabbit antibody (Cell Signaling Technology, clone D14E12, #8242, 1:200)<br>Alexa Fluor 594-conjugated F(ab')2 fragments of goat anti-rabbit IgG (H+L) (Thermo Fisher Scientific, #1:1000)<br><br>(Immunofluorescence)<br>anti-mouse CD3ε (BD Pharmingen, clone 500A2, #553239, 1:25), anti-mouse B220 (eBioscience, clone RA3-6B2, # 13-0452, 1: 50), anti-mouse CD11c (eBioscience, clone N418, # 13-0114-85, 1: 50),<br>anti-human CD68 (eBioscience, clone 815CU17, #13-0687, 1: 50), anti-human CD19 (abcam, clone EPR5906, #ab134114, 1:100),<br>anti-human IgA (SouthernBiotech, polyclonal, # 2052-31, 1: 100)
Validation	All antibodies from commercial vendors were validated by the manufacturers on their websites.

Eukaryotic cell lines

Policy information about cell lines

Cell line source(s)	MC38 (murine colon adenocarcinoma) cell line was provided by James P. Allison of the Memorial Sloan Kettering Cancer Center.<br>Jurkat, clone E6-1 was obtained from Art Weiss(UCSF, CA, USA).
Authentication	The cell lines were not authenticated
Mycoplasma contamination	MC38 cell lines were tested, and they were mycoplasma free. Jurkat cell lines were not tested.
Commonly misidentified lines<br>(See ICLAC register)	No commonly misidentified cell lines were used.

Animals and other organisms

Policy information about studies involving animals; ARRIVE guidelines recommended for reporting animal research

Laboratory animals	WT (C57BL/6J or C57BL/6N), muMt-/- mice (C57BL/6J), Cd3e-/- mice (C57BL/6J), rag1-/-mice (C57BL/6J), mb1cre/+- gad1fl/+ and mb1cre/+- gad1fl/fl mice were used in this study. Both male and female mice were used. 2 Mo-5 Mo old mice were used in this study. The SPF facility of RIKEN is maintained in a 12-hour light, 12-hour dark cycle at 23 ± 2 °C with 50 ± 10% humidity.
Wild animals	The study did not involve wild animals.
Field-collected samples	The study did not involve samples collected from the field.

Ethics oversight	All experiments were conducted in accordance with protocols approved by the Institutional Animal Care and Use Committee of the RIKEN Yokohama Branch.

Note that full information on the approval of the study protocol must also be provided in the manuscript.

Human research participants

Policy information about studies involving human research participants

Population characteristics	For blood cell study, healthy volunteers of male and female and age 20-40 with various genetic background were participated. For RA study, Japanese female diagnosed as RA from age 31-75 were participated.
Recruitment	The students and researchers in Kyoto university were recruited as the healthy volunteers through the oral announcement of the study. Since the participants were age 20-40, it may affect the interpretation of the results in terms of generalization to a broader age. Rheumatoid arthritis patients were enrolled in the Kyoto University Rheumatoid Arthritis Management Alliance (KURAMA) cohort and all were Japanese. The genetic background may affect the interpretation of the result.
Ethics oversight	All experiments were conducted in accordance with protocols approved by ethical committee of RIKEN.

Note that full information on the approval of the study protocol must also be provided in the manuscript.

Clinical data

Policy information about clinical studies

All manuscripts should comply with the ICMJE guidelines for publication of clinical research and a completed CONSORT checklist must be included with all submissions.

Clinical trial registration	Kyoto University Rheumatoid Arthritis Management Alliance (KURAMA) cohort
Study protocol	http://allie.dbcls.jp/pair/KURAMA;Kyoto+University+Rheumatoid+Arthritis+Management+Alliance.html
Data collection	The description was provided in the manuscript.
Outcomes	The description was provided in the manuscript.

Flow Cytometry

Plots

Confirm that:

☒ The axis labels state the marker and fluorochrome used (e.g. CD4-FITC).

☒ The axis scales are clearly visible. Include numbers along axes only for bottom left plot of group (a 'group' is an analysis of identical markers).

☒ All plots are contour plots with outliers or pseudocolor plots.

☒ A numerical value for number of cells or percentage (with statistics) is provided.

Methodology

Sample preparation	Mice cells were prepared from spleen, lymph node(LN), small intestine(SI) and bone marrow(BM). For making single cell suspension, spleen and LN were mashed, BM cells were flushed out using syringe and needles from femurs and tibiae, and SI was digested with collagenase (1.5 mg/ml; 30 min twice). Tumor tissues were minced and digested with collagenase (1.5 mg/ml; 30 min once).
Instrument	BD Aria II flow cytometry system was used for collection of FACS data
Software	Flowjo software (10.7.1) was used for FACS analysis.
Cell population abundance	More than 200,000 cells of the targeted populations were sorted using the high purity mode.
Gating strategy	Using the FSC/SSC gating, debris was removed, and the single alive cells were gated. Each population was gated based on the surface or intracellular markers as described in the manuscript.

☒ Tick this box to confirm that a figure exemplifying the gating strategy is provided in the Supplementary Information.
